# Media Screening for Obtaining *Haematococcus pluvialis* Red Motile Macrozooids Rich in Astaxanthin and Fatty Acids

**DOI:** 10.3390/biology7010002

**Published:** 2017-12-26

**Authors:** Thomas O. Butler, Gordon J. McDougall, Raymond Campbell, Michele S. Stanley, John G. Day

**Affiliations:** 1 Department of Biological and Chemical Engineering, Sheffield University, Sheffield S1 3JD, UK; 2 Scottish Association for Marine Science, Scottish Marine Institute, Oban PA37 1QA, UK; michele.stanley@sams.ac.uk (M.S.S.); John.Day@sams.ac.uk (J.G.D.); 3 The James Hutton Institute, Invergowrie, Dundee DD2 5DA, UK; gordon.mcdougall@hutton.ac.uk (G.J.M.); raymond.campbell@hutton.ac.uk (R.C.)

**Keywords:** algal biotechnology, astaxanthin, carotenoids, fatty acids, *Haematococcus*, red motile macrozooids

## Abstract

Astaxanthin from *Haematococcus pluvialis* is commercially produced in a two-stage process, involving green vegetative (macrozooid) and red aplanospore stages. This approach has been scaled up to an industrial process but constraints limit its commercial success and profitability, including: contamination issues, high pigment extraction costs, requirements for high light levels and photo-bleaching in the red stage. However, in addition to the aplanospore stage, this alga can produce astaxanthin in vegetative palmelloid and motile macrozooid cells. In this study, a two-stage process utilising different media in the green stage, with subsequent re-suspension in medium without nitrate was employed to optimise the formation of red motile macrozooids. Optimal growth in the green phase was obtained on cultivation under mixotrophic conditions in EG:JM media followed by re-suspension in medium without nitrate resulting in red motile macrozooids with an astaxanthin content of 2.74% (78.4% of total carotenoids) and a lipid content of 35.3% (rich in unsaturated fatty acids. It is envisaged that the red motile macrozooids could be harvested and fed as a whole-cell product directly in the animal feed and aquaculture sectors, or used as a blend of carotenoids and polyunsaturated fatty acids (PUFAs) in nutraceutical products.

## 1. Introduction 

Astaxanthin is a high value keto-carotenoid synthesised from ß-carotene by the introduction of hydroxyl and keto-moieties at the 3,3’ and 4,4’ positions of the ß-ionone rings. The oxygenated groups make keto-carotenoids relatively more polar, allow esterification and can lead to a higher antioxidant activity [1]. Astaxanthin is found in many organisms through dietary intake but the primary producers of this carotenoid are limited; a range of microorganisms and plants e.g., bacteria, algae, fungi and members of the *Adonis* genus in higher plants (Table S1, Supplementary Materials). To date, the green alga *Haematococcus pluvialis* (Chlorophyta, Volvocales) has the highest reported level of astaxanthin at 4% dry weight (DW) [2]. Furthermore, the purity of astaxanthin produced by *H. pluvialis* is much higher than other microalgae and can reach 95% of the total carotenoids [3,4]. Most studies report a carotenoid composition of 85% astaxanthin [5,6]. Thus, this alga has been extensively studied and is the organism of choice for those developing commercial-scale processes [7,8,9,10,11].

The market value of astaxanthin is expected to exceed $1.5 billion by 2020 [12], mainly incorporated in dietary supplements, nutraceuticals, cosmetics, as well as feed additives in the aquaculture and agriculture sectors [5,13,14]. Currently over 95% of astaxanthin utilised for these applications is chemically synthesised, with <1% derived from *H. pluvialis* [15]. To a large extent this is due to the cost of production, as synthetic astaxanthin is around $1000/kg, compared to *H. pluvialis* derived astaxanthin at ~$3000–$3600/kg [16,17]. However, concerns have been raised linked to the sustainability of synthetic astaxanthin production as it is derived from petrochemicals [17]. Also, the stereochemistry differs between the synthetic and *H. pluvialis* derived forms with the (3S, 3’S) form predominant in *H. pluvialis* and a mixture of the three stereoisomers (3R, 3’R), (3R, 3’S) and (3S, 3’S) in ratios of 1:2:1 in synthetically synthesised material [18]. There are also concerns about efficacy and human health benefits as it has been reported that the isomer found in *H. pluvialis* has a higher bioactivity, compared to synthetic astaxanthin [1,6,13]. Additionally, this pigment is accepted as a natural product, has been approved as a colour additive for salmon feeds and as a nutraceutical for human use in the USA, Japan and several European countries [19]. Furthermore, the US Food and Drug Administration (FDA) has granted astaxanthin from *H. pluvialis* “GRAS status” (generally regarded as safe) [6,20].

Commercial production of *H. pluvialis*-derived astaxanthin has involved a two-stage culture system with a green stage, for maximal biomass production and a red stage, for maximising astaxanthin production [2,21,22]. In outdoor two-stage production processes astaxanthin yields can reach 8–10 mg·L^−1^·day^−1^ over a 10-day cycle (4-day green stage and 6-day red stage) with astaxanthin accounting for up to 4% DW under high light and nitrate deplete conditions in the red stage [2]. This astaxanthin accumulates in the cytoplasmic oil globules acting as a photoprotective pigment under adverse conditions [23].

**Figure 1:**
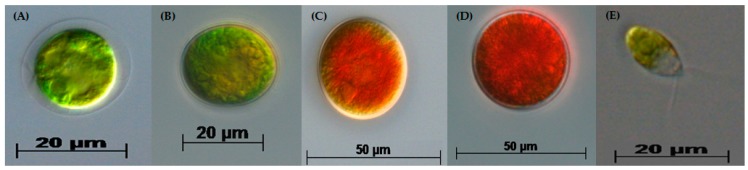
Life cycle stages of *H. pluvialis*: (**A**) green motile macrozooid, (**B**) early stage palmelloid, (**C**) late-stage palmelloid, (**D**) aplanospore (haematocyst) and (**E**) green motile microzooid.

*H. pluvialis* has a complex life cycle with three common morphotypes observed; green motile macroozoids, palmelloids and aplanospores (Figure 1) [24]. In production systems, accumulation of astaxanthin conventionally occurs in aplanospores and is induced by growth-limiting conditions with deprivation of nutrients and/or exposure to high light [25,26]. However, aplanospores provide poor astaxanthin bioavailability when ingested directly due to the presence of a thick sporopollenin cell wall and feeding trials with intact aplanospores did not result in pigmentation in salmonids [27,28]. Disruption of these cell walls has proven difficult even when using harsh treatments including acetolysis and autoclaving, which will invariably result in losses of astaxanthin [29,30,31]. Extraction using supercritical CO_2_ is the method of choice, with a low temperature and pressure (31.1 °C and 1085 psi) [32], resulting in release and stabilisation of the pigment. However, the equipment needed is expensive with high capital and operational costs [33].

Although there are a significant number of commercial plants producing algal-derived astaxanthin, process development has been challenging. Each stage requires optimisation to maximise biomass (Phase 1) and pigment production (Phase 2). Additionally, *H. pluvialis* is susceptible to contamination in both the green and red stages. The majority of commercial producers utilise closed photobioreactors (Table S2, Supplementary Materials) employing two-stage processes, as large-scale, single-phase, open pond systems have proved unsatisfactory, primarily due to difficulties with contamination [34,35]. A novel pathogen, the chytrid *Paraphysoderma sedebokerensis*, which is closely related to the plant pathogen *Physoderma* has recently been characterised and has a 100% infection rate of *H. pluvialis* in 3–4 days [36,37]. Palmelloid and aplanospore morphotypes of *H. pluvialis* have been reported to be infected but motile macrozooids remain uninfected [36,38]. This has been described as the most serious hurdle to commercial success, being responsible for reductions in astaxanthin productivities and frequent culture collapse [13]. Mass cell die-off (photo-bleaching) has also been noted in the transition between the green and red stages of the process, where the cell density can decrease by 41% [39]. The causes of cell death on transfer from the green to the red stage of cultivation are not as yet fully elucidated [40] but reduction of “die-off” between the stages is crucial to minimise losses in productivity.

A continuous, one-stage astaxanthin production process has been developed with aims of increasing productivity, using a mixed culture of motile macrozooids and palmelloids/aplanospores [41]. This one-stage process has been reported to produce 20.8 mg·L^−1^·day astaxanthin (Table S2, Supplementary Materials), formed under nitrate deficient conditions [42]. However, the maximum astaxanthin content obtained was 0.8% DW [42] with a low purity of astaxanthin (65% of total carotenoids) [43].

The aim of this study was to cultivate *H. pluvialis* in different autotrophic and mixotrophic media to maximise biomass levels in the green stage, with characterisation of astaxanthin and fatty acids in red motile macrozooids in the red stage under optimal conditions. 

## 2. Materials and Methods

### 2.1. Microalgal Culture and Purification Procedure

*Haematococcus pluvialis* SAG 34/1d was obtained from the Culture Collection of Algae at Göttingen University (SAG, Goettingen, Germany). The alga was re-cloned and treated with antibiotics to ensure axenicity [44]. In brief, Bold Basal Medium (BBM) with three-fold nitrogen and vitamin (3N-BBM+V) agar plates (www.CCAP.ac.uk) were prepared with 0.5 mg·mL^−1^ ampicillin and 0.1 mg·mL^−1^ cefotaxime. The antibiotics were filter sterilised through a 0.22 µm polyethersulphone (PES) filter (JetBiofil, Guangzhou, China) and added after the medium was sterilised by autoclaving and allowed to cool to ~50 °C. Agar plates were inoculated with the original culture and incubated at 15 °C, under a 12:12 h light:dark regime at 40 µmol photons m^2^·s^−1^ for four weeks. Single colonies formed were re-inoculated onto 3N-BBM+V agar and transferred through two successive sub-culture transfers, as outlined above. A single *H. pluvialis* colony was then inoculated into a flask containing 20 mL of 3N-BBM+V liquid medium and incubated under the above environmental regime with manual agitation. After two weeks, a 10% (*v/v*) inoculum was aseptically transferred to 250 mL flasks containing 100 mL 3N-BBM+V. These cultures were incubated on a reciprocal orbital shaker at 150 rpm (Innova 44, New Brunswick Scientific, Edison, NJ, USA) under the above standard environmental regime. Cultures (10% *v/v* inoculum) were transferred weekly to ensure availability of a standardised, vigorous, green-stage inoculum for experimental use.

### 2.2. Investigating the Influence of Medium Formulation on Cell Density in a Two Stage Process

The effect of various autotrophic and mixotrophic media on the growth of *H. pluvialis* in the green stage and red stage were assessed as outlined below. Media formulations (100 mL) of 3N-BBM+V; 3N-BBM+V + 10 mM SA; FM:FB; BG-11 and EG:JM (www.CCAP.ac.uk) (see Tables S3 and S4 for full formulation, Supplementary Materials) were prepared and sterilised in triplicate in 250 mL flasks (Table 1). Aliquots of *H. pluvialis* from the standardised stock-culture were inoculated to provide an initial cell density of 1 × 10^4^ cells·mL^−1^ (0.06 g/L DW) and the flasks incubated at 20 °C, on an orbital shaker at 150 rpm, under a 12:12 h light:dark cycle at 40 µmol photons m^2^·s^−1^. Cell density was monitored every 2 days (as outlined below) and the cells were harvested after 12 days (Section 2.5). Harvested cells were re-suspended in 100 mL of 3N-BBM+V without nitrate (medium without nitrate) and incubated at 20 °C, under continuous light (240 µmol photons m^−2^·s^−1^) for 12 days. Pictures were recorded using an Axiocam HRc (Zeiss, Oberkochen, Germany). Cell density was monitored every 3 days. Dry weight (DW) analysis was conducted at the end of the green and red stages (Section 2.4). Carotenoid and fatty acid methyl ester (FAMES) analysis was conducted at the end of the red stage.

### 2.3. Growth Measurement (Cell Number)

At each sampling point an aliquot (1 mL) of culture was aseptically removed from each replicate flask and fixed with 1% Lugols solution (Sigma Aldrich, Welwyn Garden City, UK). Enumeration was conducted using an improved Neubauer haemocytometer (Celeromics, Cambridge, UK) under phase contrast at 400× magnification with an Axio Imager 2 microscope (Zeiss, Germany). The mean growth rate (µ) was calculated on a cell basis according to the equation below [45]:

$$µ\;\left(/d\right)=\frac{\ln\left(X2)-\ln(X1\right)}{t_{2}-t_{1}}$$

X1 and X2 in the equation represent the number of cells at the start and end of the log phase at the times t_1_ and t_2_, respectively.

Doubling time (DT) to achieve a doubling of the number of viable cells was calculated according to the following equation:

DT (d)=ln(2)/µ

### 2.4. Dry Weight (DW) Measurement

Aliquots (10 mL) of samples were filtered through a pre-dried and pre-weighed GF/C Whatman filter paper (Whatman, Maidstone, UK). Samples were subsequently “washed” adding 10 mL of distilled water to remove any adhering medium etc. Samples were then dried in an oven at 80 °C overnight, then weighed. DWs were expressed as g·L^−1^.

### 2.5. Sample Preparation for Carotenoid, Astaxanthin and Fatty Acid Analysis

After 12 days incubation in the red stage, cells were harvested by centrifugation (7000× *g* for 10 min at 16 °C). The supernatant was discarded and the pellet re-suspended in 5 mL deionised water in a 50 mL Falcon tube (Eppendorf, Stevenage, UK) and then transferred into a sterile pre-weighed 2 mL Eppendorf tube. This was then centrifuged at 18,500× *g* for 2 min at 16 °C, snap frozen in liquid nitrogen (LN), then stored at −80 °C. After freeze-drying using a Beta 1-8 LD freeze-dryer (Martin Christ, Osterode, Germany), the samples were weighed and oxygen was displaced by nitrogen addition, before being sealed, then stored in at −80 °C freezer for subsequent carotenoid and fatty acid analyses.

### 2.6. Total Carotenoid Analysis

Carotenoid analysis was conducted in low light and at low temperature because astaxanthin is vulnerable to degradation in the presence of light, higher temperatures and oxygen [46]. Total carotenoid analysis was performed employing a modified version of the method detailed by Lorenz [47]. To a 2 mL Eppendorf tube, approximately 3 mg freeze-dried material was added and re-weighed. Glass beads (0.36 g) and 0.60 mL DMSO were added and the samples were then transferred to a pre-heated water bath at 45–50 °C for 30 min with vortexing every 10 min during incubation. Then acetone (0.60 mL) was added and vortexed for 30 s. The samples were centrifuged at 5000× *g* for 2 min and then the supernatant was transferred to glass tubes. If the cell pellet was coloured the above procedure was repeated until a colourless cell pellet was obtained (usually two extractions). The volume in the glass tube was adjusted to 3 mL with acetone and inverted gently to mix. The samples were then filtered through glass wool to remove any particulates and transferred to new glass tubes. Each sample was analysed in a quartz cuvette using a Nanodrop Spectrophotometer (Thermo Scientific, Loughborough, UK) and the maximum OD was recorded in the range 471–477 nm (peak range for carotenoids) against acetone as a blank. Where readings were in the range of 0.2–1.25, samples were diluted with acetone as required.

$$\mathrm{Total}\;\mathrm{carotenoid}\;\left(\%\;\mathrm{DW}\right)=\frac{\mathrm{Absorbance}\;\mathrm{maximum}}{250}\;\times \;\frac{\mathrm{volume}\;\mathrm{of}\;\mathrm{acetone}\;\times \;\mathrm{dilution}\;\mathrm{factor}}{\mathrm{sample}\;\mathrm{weight}\;\left(\mathrm{mg}\right)}\;\times \;100$$

### 2.7. Astaxanthin Analysis by Liquid Chromatography-Mass Spectrometry (LC-MS)

Astaxanthin analysis was conducted using a 6460 Triple Quad LC/MS (Agilent, Cheadle, UK) with an Agilent G4212B 1260 photodiode array detector (PDAD), G1316A 1260 TCC column oven and a G1329B 1260 auto-sampler. The software used was a MassHunter Workstation Suite version B.04.01 (Agilent, Cheadle, UK). Samples were run in full scan mode and the diode array detector set from 200–600 nm. Dried samples were re-dissolved in ethyl acetate at 1 mg·mL^−1^ with vortex mixing, then diluted 1 in 10 with methanol and placed into UPLC filter vials (0.45 mM). Samples were separated by chromatography on a reverse-phase C30, 5 mm column (250 × 4.6 mm) coupled to a 20 × 4.6 mm C30 guard (YMC Inc., Allen Town, PA, USA). The mobile phases consisted of methanol (A), water/methanol (20/80 by volume) containing 0.2% ammonium acetate (*w/v*) (B) and tert-methyl butyl ether (C). The elution profile was 95% A, 5% B isocratic for 12 min, a step to 80% A, 5% B, 15% C at 12 min, followed by a linear gradient to 30% A, 5% B, 65% C by 30 min. A conditioning phase (30 min) was then used to return the column to the initial concentrations of A and B. Standards run were astaxanthin (Sigma, Welwyn Garden City, UK) and trans-β-Apo-8’-carotenal (Sigma, Welwyn Garden City, UK), carotenoid peaks were tentatively identified according to their triple spectra according to Britton et al. [48]. Putative IDs were supported by mass spectrometry data (Figure S1 and Table S5, Supplementary Materials) which agreed with the literature values for esters of astaxanthin [49].

### 2.8. Fatty Acid Analysis

#### 2.8.1. Direct-Derivatisation

Rapid small-scale direct-derivatisation was performed according to Slocombe et al. [50]. Lyophilised *H. pluvialis* (5 mg) was weighed into Chromacol 1.5 mL screw-top vials (Thermo Scientific, Loughborough, UK). To this material, 10 μL methyl tricosanoate (Larodan, Solna, Sweden) internal standard (5 mg/mL in hexane) and 500 μL anhydrous 1 M methanolic-HCl (Sigma, Welwyn Garden City, UK) were added. The vials were flushed with nitrogen and capped with Teflon seals and incubated at 85 °C for 2 h. After cooling at room temperature, 250 μL of 0.9% (*w/v*) KCl was added and the upper hexane phase containing the fatty acid methyl esters (FAMES) was removed to Teflon-capped tapered vials (Chromacol, UK) for Gas Chromatography-Flame Ionisation Detection (GC-FID) analysis, flushing the sample with nitrogen gas before capping. Samples were either analysed immediately, or stored under nitrogen at −80 °C. A Hamilton syringe was used in all cases to avoid “noise” from plasticisers found in plasticware.

#### 2.8.2. Gas Chromatography-Flame Ionisation Detection (GC-FID)

Samples were analysed by GC-FID (GC-2014, Shimadzu, Nagoya, Japan) according to Slocombe et al. [50]. Injections were made into a 30 m, 0.25 mm ID ZB-wax column (Phenomenex, Værløse, Denmark) using helium as carrier at 1.56 mL·min^−1^ with a split ratio of 100:1. The temperature was ramped from 160 °C to 240 °C at 4 °C min^−1^ then run isothermally at 240 °C for 10 min. Peak areas were integrated using Gas Chromatography Solution Software (Shimadzu, Japan) and quantified by reference to the internal standard when expressed as % DW. Defined FA classes such as total unsaturated FA were expressed as the sum of individual % DW values corresponding to individual FAs. Peak identities were determined using external standards: 37 FAMES, PUFA2, PUFA3 (Sigma, Welwyn Garden City, UK), methyl 9 (*Z*), 12 (*Z*) Hexadecadienoate (Larodan, Solna, Sweden) and Methyl 7 (*Z*) hexadecadienoate (Cambridge Biosciences, Cambridge, UK).

### 2.9. Data Analysis

One-way ANOVA was used for testing the effects of multiple factors. Post Hoc analysis was conducted using Tukey’s test for determining which groups were significantly different. Post Hoc analysis using the Dunnett’s test was employed to determine if treatments were different from the control. All data conformed to normality (Kolmogorov-Smirnov, *p* < 0.05) and equal variance (Levene’s, *p* < 0.05).

## 3. Results and Discussion

### 3.1. Two-Stage Process—Green Stage

**Figure 2:**
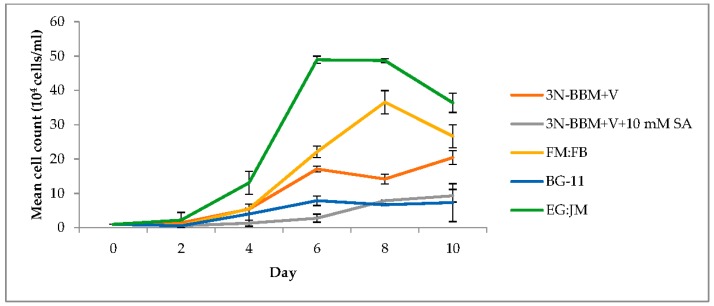
Effect of a range of media on the growth of *H. pluvialis* in the green stage. Mean cell count data ± S.D. (*n* = 3). The green stage involved culturing at 20 °C, 40 µmol photons m^−2^·s^−1^, 12:12 photoperiod, 150 rpm over a 10-day period.

Cultivation on EG:JM medium resulted in the highest cell density (3.64 × 10^5^ cells·mL^−1^) with a significantly higher yield than the other media investigated (*p* < 0.01) (Figure 2). With this medium, the maximum growth rate between 4 and 6 days was 0.66 µ/day (doubling time of 25 h). In addition to sodium acetate, this medium contains Lab Lemco powder, Tryptone and Yeast Extract, which stimulated growth more than was observed for the other mixotrophic, or photoautotrophic media tested. In a previous study, Tocquin et al. [51] reported that the N/P ratio governed the maximum growth rate; however, in this study it appeared that carbon limitation was more important in the form of yeast extract supplied by the EG:JM medium. It is also likely that tryptone was responsible for the increased growth due to an amino acid supply. Typically, a low C:N ratio results in the production of green biomass and a high C:N ratio results in astaxanthin production [52].

Using the hydroponics medium (FloraMicroBloom; FM:FB) at 1:5 mL·L^−1^ resulted in a maximum cell density of 3.66 × 10^5^ cells·mL^−1^ with maximum growth between 4 and 8 days of 0.47 µ/day (doubling time of 35 h). This is a similar growth rate to that reported by Tocquin et al. [51] when using the same FM:FB formulation. However, Tocquin et al. [51] attained a maximum cell density of 1.4 × 10^6^ cells·mL^−1^ (3.8 fold higher than this study), using the same strain of *H. pluvialis* (SAG 34/day) after 14 days. They employed a similar light regime (30 µmol photons m^−2^·s^−1^, compared with 40 µmol photons·m^−2^·s^−1^ in this study); however, the experimental design differed, with a higher initial cell density (5 × 10^4^ cells·mL^−1^) compared to 1 × 10^4^ cells·mL^−1^ and a higher incubation temperature (25 °C). 

*H. pluvialis* formed green motile macrozooids, without palmelloids, when cultivated in 3N-BBM+V and EG:JM medium. The highest biomass yield (0.41 g/L DW) was obtaining using EG:JM but this was not significantly higher than cultivation in FM:FB (*p* > 0.05).

There have been a number of reports investigating the effect of different media on maximising *H. pluvialis* biomass yield in the green stage [51,52,53,54,55,56,57,58]. Biomass production has been a major bottleneck in the two-stage process of astaxanthin production and further optimisation of the growth media is required. Commonly, BBM-based formulations have been used for *H. pluvialis* culture [51,59,60,61,62], along with BG-11 [2,43,63]. Fábregas et al. [55,64] highlighted the importance of medium formulation for microalgal productivity using optimised *Haematococcus* medium (OHM), which yielded more than three times higher biomass than with BBM (1.20 × 10^5^ cells·mL^−1^).

### 3.2. Two-Stage Process—Red Stage

**Figure 3:**
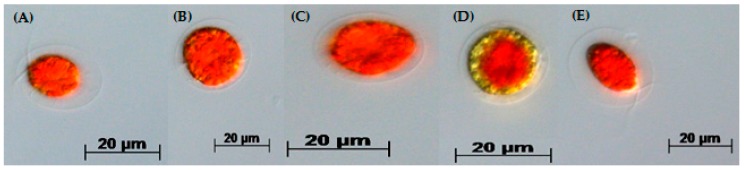
*H. pluvialis* red motile macrozooids formed in the red stage after 12 days. *H. pluvialis* cultures from the green stage were re-suspended from (**A**) 3N-BBM+V; (**B**) 3N-BBM+V + 10 mM SA; (**C**) FM:FB; (**D**) BG-11 and (**E**) EG:JM, then incubated under medium without nitrate (20 °C, continuous light at 240 µmol photons m^−2^·s^−1^). Pictures were taken with an Axio Imager 2 microscope with an Axiocam HRc.

**Figure 4:**
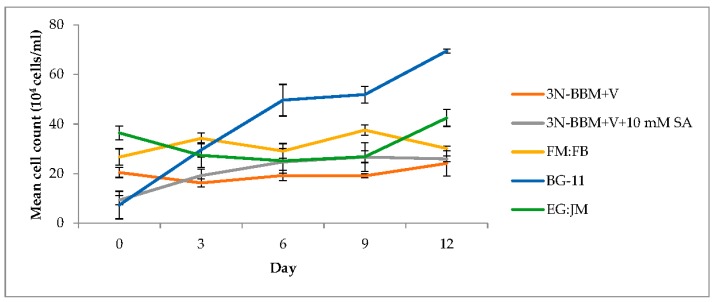
Growth of *H. pluvialis* after re-suspension of green stage culture in astaxanthin production inducting conditions. Mean cell count data ± S.D. (*n* = 3). The red stage involved culturing at 20 °C, continuous light at 240 µmol photons m^−2^·s^−1^.

**Figure 5:**
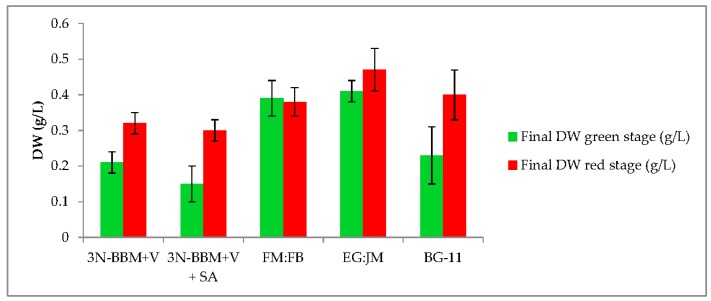
Dry weight (DW) yields of green and red stage (*H. pluvialis* cultured in a range of media under autotropic and mixotrophic conditions). Mean DW (g·L^−1^) ± S.D. (*n* = 3). Green stage involved culturing at 20 °C, 40 µmol photons m^−2^·s^−1^, 12:12 photoperiod, 150 rpm over a 10-day period. Red stage involved culturing at 20 °C, continuous light at 240 µmol photons m^−2^·s^−1^ over a 12-day period.

**Figure 6:**
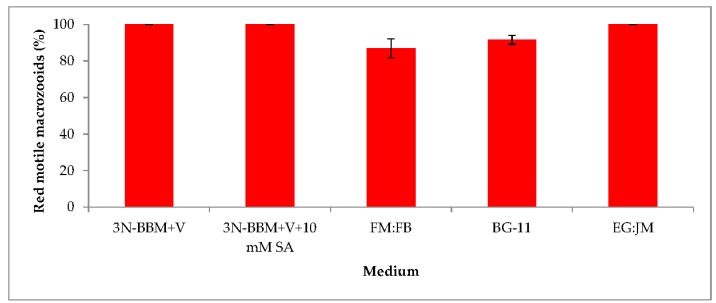
Percentage of red motile *H. pluvialis* macrozooids produced in the red stage after cultivation of *H. pluvialis* in a range of media. Mean % red motile macrozooids on day 12 ± S.D. (*n* = 3). Green stage involved culturing at 20 °C, 40 µmol photons m^−2^·s^−1^, 12:12 photoperiod, 150 rpm for 10 days. Red stage involved culturing at 20 °C, continuous light at 240 µmol photons m^−2^·s^−1^ for 12 days.

When cells from the green stage cultivated in 3N-BBM+V, 3N-BBM+V + SA and EG:JM were transferred to medium without nitrate, only red motile macrozooids were formed and no palmelloids were observed (Figure 3A,C,E). Cells cultured in BG-11 medium in the green stage and re-suspended in 3N-BBM+V without nitrate, attained the highest cell density (6.94 × 10^5^ cells·mL^−1^) after 12 days (significantly higher than in the other cultures (*p* < 0.01; Figure 4) but under these conditions a mixture of red motile macrozooids and palmelloids were formed (Figure 3D). For cultures grown in BG-11 that were subsequently transferred to the medium without nitrate, a biomass yield of 0.40 g·L^−1^ was achieved in the red stage (Figure 5), with a carotenoid content of 1.41% DW. This was lower than for the cultures grown on the other media employed in this study which were re-suspended in medium without nitrate; furthermore, these cells were clearly greener in colour (Figure 3D). Using the two-stage approach with BG-11 as the medium in the green stage resulted in a culture comprised of 92% red motile macroozoids in the red stage, with the remainder being palmelloids (Figure 6). 

**Figure 7:**
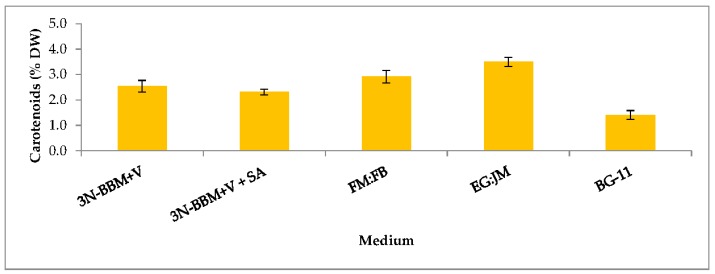
Carotenoid content (% DW) in *H. pluvialis* cultivated in a range of autotropic and mixotrophic media. Mean carotenoid content (% DW) ± S.D. (*n* = 3). Re-suspension of cultures in green stage grown in various autotrophic and mixotrophic media (10 days) in red stage induction conditions for 12 days in medium without nitrate (20 °C, continuous light at 240 µmol photons m^−2^·s^−1^).

The growth/increase in cell number observed after transferring the *H. pluvialis* cells to the medium without nitrate was assumed to be associated with nitrogen stored in the cells. In the medium used in the green phase nitrate levels were high (two-fold higher than 3N-BBM+V), with a high N/P ratio of 77:1 (Table 1). This may also have accounted for a lower carotenoid content and a lower astaxanthin accumulation (Figure 7) in these cultures. Raimbault and Mingazzini [65] reported nitrate storage in diatoms when nitrate was limited but not when nitrate concentrations were high. Furthermore, inorganic phosphate can accumulate in microalgae as polyphosphate granules that normally appear under phosphate sufficient conditions but they disappear when phosphate becomes limiting [66]. Manipulating intracellular nitrogen storage mechanisms in *H. pluvialis* may enhance biomass and astaxanthin yields and this warrants further investigation.

### 3.3. Biochemical Composition in the Red Stage

**Figure 8:**
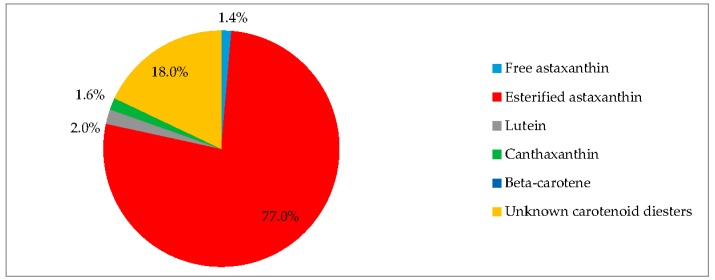
Pigment profile of *H. pluvialis* red motile macrozooids. Samples were cultured in EG:JM in the green stage, followed by medium without nitrate in the red stage (20 °C, continuous light at 240 µmol photons m^−2^·s^−1^).

EG:JM was determined to be the optimal medium for the green stage. The highest cell count and DW was obtained with cells cultured in EG:JM, then re-suspended in the medium without nitrate, with a biomass yield of 0.47 g·L^−1^ (Figure 5) with the highest carotenoid content of 3.50% DW (Figure 7). The astaxanthin content was 2.74% ± 0.11% DW and amounted to 78.4% of total carotenoids with 77% in the esterified forms (Figure 8). The astaxanthin monoesters were esterified with oleic and linoleic acids. Diesters were present but the esterified FAs were not identified. To our knowledge, this is the highest reported astaxanthin content and composition in red motile macrozooids. It was interestingly observed that 18% of the carotenoids were unknown and these would be useful to characterise in future studies.

A relatively small number of papers have reported carotenoid accumulation in red motile macrozooids (Grünewald et al. [67]; Hagen et al. [9]; Brinda et al. [68] and Tocquin et al. [51]) but only Hagen et al. [9] and Brinda et al. [68] quantified their astaxanthin content. The maximum level reported was 2% DW when cells from the green stage cultivated in BBM were re-suspended in nitrate and phosphate deplete BBM medium Brinda et al. [68], Del Río et al. [42] reported an astaxanthin content of 0.8% DW in the red stage in macrozooids and palmelloids, with 65% of the carotenoids being astaxanthin. In a subsequent study Del Río et al. [41] reported that a one stage process resulted in an astaxanthin content of 1.1% DW, with astaxanthin comprising 85% of the total carotenoids; however, this was a mixed population of red motile macrozooids and palmelloids.

To date, red motile macrozooids have only been reported in a few *Haematococcus* strains; *H. pluvialis* SAG 192.80 [9,67], *H. pluvialis* SAG 19-a [68], *H. pluvialis* CCAP 34/8 [10,41,42] and *H. pluvialis* CCAP 34/1D/SAG 34/day ([51], this study). It is possible this response is strain specific and/or induced by the medium. Although it has not been fully elucidated what governs the formation of red motile macrozooids, medium composition and the N/P ratio play an important role. When green stage cultivated cells were re-suspended in different 3N-BBM+V formulations (astaxanthin induction media), only those without nitrate, phosphate, nitrate and phosphate and nitrate deprived medium (0.88 mM) resulted in the accumulation of red motile macrozooids (Figure S1, Supplementary Materials).

**Figure 9:**
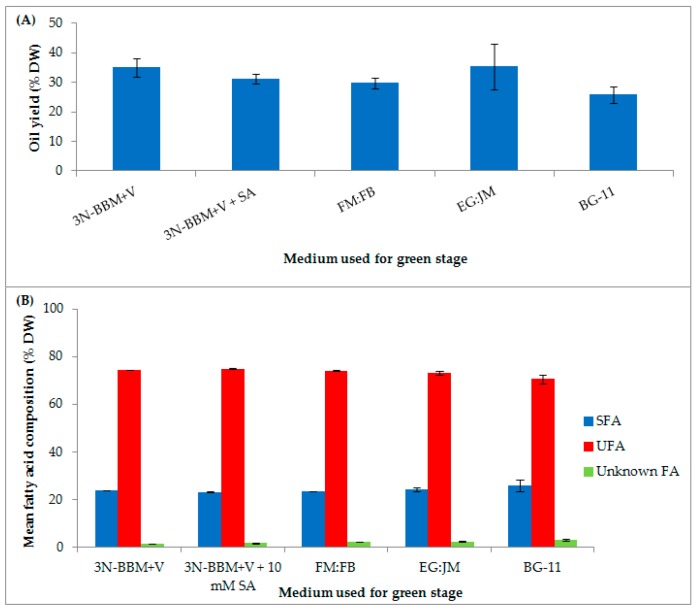
*H. pluvialis* cultivated in a range of media under autotropic and mixotrophic conditions (**A**) Mean total fatty acid content (% DW); (**B**) mean fatty acid composition (% DW) as saturated, unsaturated or unknown fatty acids. Mean DW (g·L^−1^) ± S.D. (*n* = 3). Cultures incubated for 10 days in the green stage in various autotrophic and mixotrophic media and 12 days in the red stage in medium without nitrate (20 °C, continuous light at 240 µmol photons m^−2^·s^−1^). SFA = Saturated fatty acid, UFA = Unsaturated fatty acid, Unknown FA = Unknown fatty acid.

In addition to pigment production *H. pluvialis* accumulates high levels of lipids, up to 35% DW [61]. In this study, the total fatty acid (TFA) content ranged from 25–35% DW depending nutrient regime (Figure 9A). The highest TFA content (35.32 ± 7.77% DW) was in *H. pluvialis* cultured in EG:JM in the green stage, then transferred to the standard astaxanthin production conditions in the red stage.

The proportion of unsaturated and saturated FAs of TFAs in *H. pluvialis* cultured in this study remained similar irrespective of the medium used for the green stage (Table 2). Unsaturated fatty acids (UFAs), accounted for >70% of the FA composition with saturated fatty acids (SFAs) accounting for between 23–26% of the FA composition (Figure 9B). On cultivation under mixotrophic conditions, UFAs accounted for 73.20 ± 0.66% of the FA composition when *H. pluvialis* cultured in EG:JM in the green stage was re-suspended in astaxanthin inductive conditions in the red stage. The most abundant FAs were linoleic (18:2 (n-6)), palmitic (16:0) and oleic (18:1 (n-9)) respectively (Table 2). In a previous study employing a one-stage process, when nitrate was limited to 1.7 mM, formation of macrozooid and palmelloids, with a fatty acid content of 7.6% DW was observed [69]. Haematocysts (aplanospores) generated in the two-stage process, under nitrogen starvation accumulated only 3.3% DW FAs [69]. However, Damiani et al. [61] reported lipid levels up to 35% DW in *H. pluvialis* aplanospores cultivated for 14 days in nitrate deplete BBM, which are comparable to the levels observed in this study in red motile macrozooids.

### 3.4. Red Motile Macrozooids—Rich in PUFAs and Astaxanthin

Currently the production of biofuels from microalgae is non-profitable [70,71] but the rise in fossil fuel prices, depletion of world reserves of fuel and climate change have resulted in sustained interest for microalgal biofuels. Furthermore, the investment in research into algal biofuels has stimulated the development of a variety of commercially viable products particularly in the nutraceutical and cosmetic sectors [72]. Additionally, increased emphasis has been placed on maximising value from any process and the use of a biorefinery approach, where primary products are supplemented by additional use of the material to generate several products [73]. In this study, *H. pluvialis* produced commercially interesting levels of both astaxanthin and lipids and this raises a potential opportunity for further development in a biorefinery context. Using EG:JM in the green stage followed by re-suspension in medium without nitrate in the red stage resulted in a population of red motile macrozooids without palmelloids or aplanospores. Here the cells had an astaxanthin content of 2.74% DW astaxanthin (78.4% of total carotenoids) with a total FA content of 35.32% DW with high levels of UFAs (73.2%).

To date only aplanospores have been assayed for fatty acid analysis and only a few studies have focused on *H. pluvialis* as a source of lipids or biofuel [61,74,75,76]. This is the first report that has characterised the fatty acid analysis of *H. pluvialis* red motile macrozooids. The high levels of UFAs in *H. pluvialis* suggest that it has potential applications in higher value food and nutraceutical products rather than as a biofuel as previously suggested [61]. The red motile macrozooids could be used as a whole cell product for aquaculture, providing digestibility studies were conducted and sufficient pigmentation of salmonids was observed. Additionally, as *H. pluvialis* is GRAS certified, the cells could be directly formulated in human products as a nutraceutical, as esterified astaxanthin has a high antioxidant content [69] and in this study up to 98% of the astaxanthin was esterified as diesters and monoesters. Both these esterified forms of astaxanthin can contribute to the antioxidant activity [69]. Furthermore, these thin-walled cells could also enhance bioaccessibility and bioavailability of both lipids and carotenoids. 

## 4. Conclusions and Future Research Direction

The red motile macroozooids generated in this study may have further biotechnological advantages over previously produced morphotypes such as aplanospores. Further investigation is warranted into whether the red motile macrozooids can be infected by chytrids. If they are not infected, this could address the major threat to the industry that infection poses. Additionally, these thin-walled cell morphotypes may hold promise for genetic engineering for the production of mutants with increased levels of astaxanthin without the requirement for the expensive and technically challenging biolistic particle delivery system for transformation.

## Figures and Tables

**Table 1:** Nitrate, phosphate and N/P ratio of media used in green stage cultivation.

Composition	3N-BBM+V	3N-BBM+V + SA	BG-11	EG:JM	FM:FB
NO_3_ (mM)	8.82	8.82	17.65	0.47	2.70
PO_4_ (mM)	1.72	1.72	0.23	0.096	4.60
N/P ratio	5.13	5.13	76.74	4.90	0.59:1

**Table 2:** Mean fatty acid composition % total ± S.D. (% TFA) of *H. pluvialis* cultivated in various autotrophic and mixotrophic media.

Fatty Acid	3N-BBM+V	3N-BBM+V + 10 mM SA	FM:FB	EG:JM	BG-11
14:0	0.35 ± 0.01	0.33 ± 0.00	0.36 ± 0.01	0.54 ± 0.15	0.43 ± 0.04
16:0	22.12 ± 0.08	21.35 ± 0.26	21.65 ± 0.08	22.57 ± 0.62	21.98 ± 0.30
16:3(n-3)	2.09 ± 0.08	2.16 ± 0.12	2.14 ± 0.05	1.86 ± 0.05	2.03 ± 0.08
16:4(n-3)	3.53 ± 0.03	4.08 ± 0.15	4.34 ± 0.41	5.37 ± 0.46	4.09 ± 0.21
18:1(n-7)	3.72 ± 0.03	3.76 ± 0.16	4.08 ± 0.27	4.76 ± 0.45	4.17 ± 0.14
18:1(n-9)	20.31 ± 0.30	18.37 ± 1.09	17.30 ± 1.42	12.31 ± 0.79	19.47 ± 0.99
18:2(n-6)	28.49 ± 0.21	28.26 ± 1.23	27.38 ± 0.66	23.63 ± 0.54	25.91 ± 0.32
18:3(n-3)	10.98 ± 0.05	12.35 ± 0.44	13.05 ± 0.90	15.45 ± 1.51	11.96 ± 0.37
18:4(n-3)	1.61 ± 0.03	1.95 ± 0.08	2.14 ± 0.26	2.74 ± 0.27	1.75 ± 0.06
Other SFA	1.39 ± 0.03	1.41 ± 0.05	1.32 ± 0.04	2.74 ± 1.57	1.77 ± 0.31
Other UFA	3.53 ± 0.07	3.97 ± 0.10	3.68 ± 0.34	4.55 ± 0.26	3.73 ± 0.07
Unknown FA	1.67 ± 0.04	1.84 ± 0.07	2.35 ± 0.12	3.27 ± 0.54	2.45 ± 0.21

Cultures incubated for 10 days in the green stage followed by subsequent red stage induction (20 °C, continuous light at 240 µmol photons m^−2^·s^−1^) for 12 days.

## Supplementary Materials

The following are available online at www.mdpi.com/2079-7737/7/1/2/s1, Table S1: Sources of astaxanthin from natural sources, Table S2: *H. pluvialis* biomass and astaxanthin productivity, Table S3: Chemical composition of autotrophic and mixotrophic media used in the initial medium preference study for the green stage, Table S4: Composition of FM:FB. Nutrient concentrations (guaranteed minimum concentrations) in mg·L^−1^ calculated from GHE, France, Figure S1: Identification of carotenoid peaks by Liquid Chromatography-Mass Spectrometry (LC-MS), Table S5: Liquid Chromatography Mass Spectrometry (LC-MS) data, Figure S2: Effect of nitrate and phosphate concentration on growth in *H. pluvialis*.
