# Supplementary material for: Media Screening for Obtaining Haematococcus pluvialis Red Motile Macrozooids Rich in Astaxanthin and Fatty Acids
Source: Biology (Basel). 2017 Dec 26;7(1):2. doi: 10.3390/biology7010002 (PMC5872028; doi:10.3390/biology7010002)
Supplement: Supplementary file 1 [file biology-07-00002-s001.pdf]

Media screening for obtaining *Haematococcus pluvialis* red motile macrozooids rich in astaxanthin and fatty acids

Thomas O. Butler, Gordon J. McDougall, Raymond Campbell, Michele S. Stanley, and John G Day

Supplementary information

Table S1 Sources of astaxanthin from natural origins

**Species**	**Class**	**Astaxanthin content (% DW)**	**Reference**
Haematococcus pluvialis	Chlorophyceae	4.0	[2]
Chlorella zofingiensis	Chlorophyceae	0.7	[77]
Scenedesmus vacuolatus	Chlorophyceae	0.3	[77]
Scotiellopsis oocystiformis	Chlorophyceae	1.1	[77]
Neochloris wimmeri	Chlorophyceae	1.9	[77]
Protosiphon botryoides	Chlorophyceae	1.4	[77]
Chlorococcum sp.	Chlorophyceae	0.2	[78]
Adonis annua	Dicotyledons	1	[79]
Ulva intestinalis	Ulvophyceae	0.02	[80]
Ulva lactuca	Ulvophyceae	0.01	[80]
Catenella repens	Florideophyceae	0.02	[80]
Agrobacterium aurantiacum	Alphaproteobacteria	0.01	[81]
Paracoccus carotinifaciens	Alphaproteobacteria	2.2	[82]
Phaffia rhodozyma/ Xanthophyllomyces dendrorhous	Tremellomycetes	0.01	[83]

Table S2 *H. pluvialis* biomass and astaxanthin productivity

**Strain no.**	**Conditions**	**Culture time (days)**	**Biomass yields green stage (g L^{-1} DW)**	**Biomass productivity green stage (g L^{-1} d^{-1} DW)**	**Astaxanthin content red stage (mg g^{-1} DW)**	**Astaxanthin yield red stage (mg L^{-1} DW)**	**Astaxanthin productivity green + red stages (mg L^{-1} d^{-1} DW)**	**Ref.**
CCAP 34/7	30 L air-lift bioreactor (autotrophic)	90	1.6	0.02	27	43.2	0.5	[27]
UTEX 16	3.7 L Fed-batch bioreactor (mixotrophic)		2.74	0.14	43	64.4	3.2	[84]
NIES-144	2.3 L Maxblend fermentor. pH stat fed-batch (sequential heterotrophic-autotrophic)	30 (20+10)	7	0.35	98	114	3.8	[85]
CCAP 34/8	50 L tubular bioreactor, 0.6 mM nitrate for red stage, continuous one-stage production process outdoors (autotrophic)		0.7	0.6-0.7	6-13	11	3.5-8	[56]
Unknown strain from Aquasearch	Green stage in Aquasearch growth modules (AGMs), followed by open pond astaxanthin induction (autotrophic)	19 (14+5)	0.2-0.36	0.036-0.052	28-30		2.2	[22]
CCAP 34/8	2 L jacked bubble column bioreactor, 2.7 mM nitrate deprivation in red stage, one-stage continuous process indoors (autotrophic)		1.03	0.93	6	6.2	5.6	[43]
NIES-144	Astaxanthin induction using nitrate depletion, with 5 % CO_2 (autotrophic)	28	2.27	0.08	77.2	175.7	6.3	[86]

Strain no.	Conditions	Culture time (days)	Biomass yields green stage (g L^{-1} DW)	Biomass productivity green stage (g L^{-1} d^{-1} DW)	Astaxanthin content red stage (mg g^{-1} DW)	Astaxanthin yield red stage (mg L^{-1} DW)	Astaxanthin productivity green + red stages (mg L^{-1} d^{-1} DW)	Ref.
CCAP 34/8	2L jacketed bubble column bioreactors, one-stage continuous process indoors (autotrophic)			1.9	11		20.8	[42]*
CCAP 34/8	55 L tubular bioreactor, nitrate reduced to < 5 mM for astaxanthin accumulation (autotrophic)	16	7	0.55	20	72	4.5	[87]
SAG 34/1b	1 L cylindrical air-lift double-region bioreactor modified into cone shape (inner core green stage, outer core red stage), batch production (autotrophic)	22 (12+10)	4 (inner core) and 7 (outer core) after day 12 FW**	0.33 FW	49.5	357	16.2	[58]
SCCAP k-0084	500 mL glass columns, two-stage strategy indoors	10 (4+6)	2	0.5	40	115	11.5	[2]
	500 L flat vertical plate bioreactor (green stage), 2000 L tubular bioreactor (red stage), two stage strategy outdoors	10 (4+6)	1.48	0.37	40	101	10.1	
NIES-144	Bubble column fed-batch process (autotrophic)	55 (12.5+ 42.5)	6.7	0.2	36	390	7.2	[88]
NIES-144	1 L flat panel bioreactor with stepwise increase in irradiance, astaxanthin induction using 5 % CO_2 and high light (autotrophic)	13.5 (4.5+9)	1.47	0.33	47	190	14.1	[89]

Strain no.	Conditions	Culture time (days)	Biomass yields green stage (g L^{-1} DW)	Biomass productivity green stage (g L^{-1} d^{-1} DW)	Astaxanthin content red stage (mg g^{-1} DW)	Astaxanthin yield red stage (mg L^{-1} DW)	Astaxanthin productivity green + red stages (mg L^{-1} d^{-1} DW)	Ref.
WBG 26	Double-layer circular pond, two-step one-stage process (autotrophic)	12	1.83	0.15	27.9	51.1	4.3	[62]
NIES-144	3 L fermentor with stepwise increase in irradiance in fed-batch and perfusion processes (mixotrophic)							[90]
	Batch	26 (6+20)	0.96	0.16	49	213	8.2	
	Fed-batch	30 (10+20)	2.8	0.28		437	15.1	
	Perfusion	38 (14+24)	4.48	0.32		602	15.8	
SKLBE ZY-18	Sequential Heterotrophy-Dilution-Photoinduction (SHDP)	27 (17 + 10)	26	1.54	46	64	6.4	[91]

*The publication by Del Río et al. [42] reported the highest astaxanthin productivity (mg $L^{-1}d^{-1}$) to date using the one-stage continuous production process

**FW = Fresh weight

Table S3 Chemical composition of autotrophic and mixotrophic media used in the initial medium preference study for the green stage

Medium component	3N-BBM+V	3N-BBM+V+ SA	BG-11	EG:JM
$NaNO_3$ (mgL^{-1})	750	750	1500	40
Na_2HPO_4 (mgL^{-1})				
K_2HPO_4 (mgL^{-1})	75	75	40	
KH_2PO_4 (mgL^{-1})	175	175		6.2
Na_2HPO_4 $12H_2O$ (mgL^{-1})				18
$MgSO_4$ $7H_2O$ (mgL^{-1})	75	75	75	25
$C_6H_8O_7$ (mgL^{-1})			6	
Ammonium ferric citrate green (mgL^{-1})			6	
$Ca(NO_3)_2$ $4H_2O$ (mgL^{-1})				10
$CaCl_2$ $2H_2O$ (mgL^{-1})	25	25	36	
$CaCl_2$ (mgL^{-1})				5

$FeCl_2\ 6H_2O$ (mgL^{-1})	0.582	0.582		
$CoCl_2\ 6H_2O$ (mgL^{-1})	0.012	0.012		
$ZnCl_2$ (mgL^{-1})	0.03	0.03		
$Co(NO_3)_2\ 6H_2O$ (mgL^{-1})			0.05	
$CuSO_4\ 5H_2O$ (mgL^{-1})			0.08	
Cr_2O_3 (mgL^{-1})				
$MnCl_2\ 4H_2O$ (mgL^{-1})	0.246	0.246	1.81	0.695
Na_2MoO_4 (mgL^{-1})				
$Na_2MoO_4\ 2H_2O$ (mgL^{-1})	0.024	0.024	0.39	
$(NH_4)_6Mo_7)_{24}\ 4H_2O$ (mgL^{-1})				0.5
H_3BO_3 (mgL^{-1})			2.86	1.24
$ZnSO_4\ 7H_2O$ (mgL^{-1})			0.22	
Na_2CO_3 (mgL^{-1})			20	
Na_2-EDTA (mgL^{-1})			1	1.125
FeNa-EDTA (mgL^{-1})				1.125
$C_2H_3NaO_2$ (mgL^{-1})		820		240
Lab-Lemco powder (mgL^{-1})				500
Tryptone (mgL^{-1})				1000
Yeast extract (mgL^{-1})				1000
NaCl (mgL^{-1})	25	25		
$NaHCO_3$ (mgL^{-1})				7.95
Thiamine-HCl (mgL^{-1})	0.012	0.012		0.02
B_{12} (mgL^{-1})	0.01	0.01		0.02

Table S4 Composition of FM:FB. Nutrient concentrations (guaranteed minimum concentrations) in mg L^{-1} calculated from information from GHE, France

**Constituent**	mgL^{-1}
Nitrogen as ammonium	10
Nitrogen as nitrate	40
Phosphate (P_2O_5)	250
Potassium (K_2O)	200
Magnesium	250
Sulphur	50
Boron	0.1
Calcium	70
Copper	0.1
Iron	1.2
Manganese	0.4
Molybdenum	0.04
Zinc	0.15

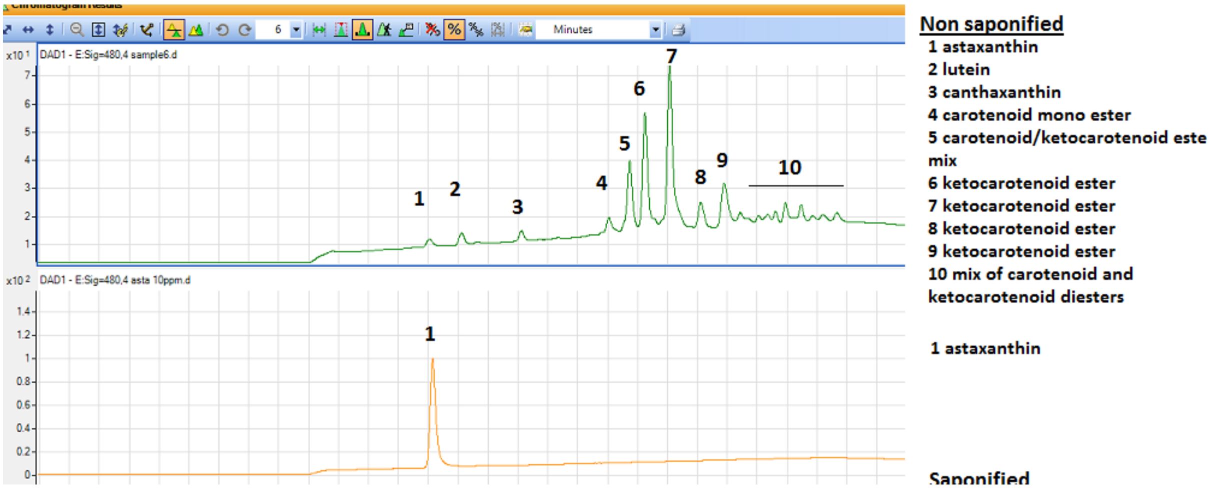

Figure S1 Identification of carotenoid peaks by Liquid Chromatography-Mass Spectrometry (LC-MS)

Table S5 Liquid Chromatography Mass Spectrometry (LC-MS) data

Peak No	*m/z* [M+H]	Putative Identity
1	619	Astaxanthin[a]
2	591	Lutein[a]
3	**565**, 588 (+Na)	Canthaxanthin[a]
4	864.7	Unknown Astaxanthin monoester[b]
5	864.7	Unknown Astaxanthin monoester[b]
6	859.6	Astaxanthin monoester (18:2)
6a	866.7	+ 2 amu >than peaks 4 & 5
7	**861.6**, 883.6 (+Na)	Astaxanthin monoester 18:1
8	898.9	Unknown Astaxanthin ester
9	868.7	Unknown Astaxanthin ester
10	870.7, 872.7, 874.8, 876.8	Mix of esterified forms

Values in bold are major signals.

a – identity confirmed by standards

b – possibly astaxanthin monoester (14:0 + NH_3) using data from Murphy et al. [92]

Most IDs are from Holtin et al. [49] and Frassanito et al. [93]

The main peaks 6 & 7 actually match with the relative abundance shown in this paper. The more hydrophobic peaks after peak 9 are probably diesters which do not yield detectable M+H ions, possibly due to multiply charged forms.

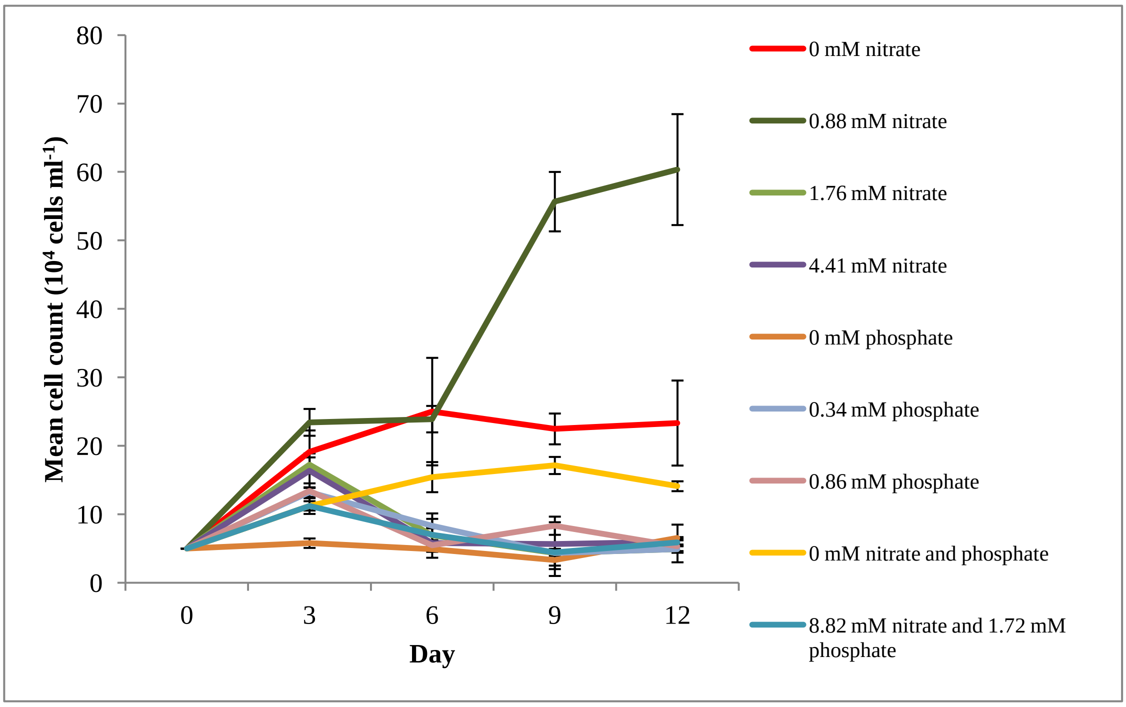

Figure S2 Effect of nitrate and phosphate concentration on growth in *H. pluvialis* (n = 3)

References

2. Aflalo, C.; Meshulam,. Y.; Zarka, A.; Boussiba, S. On the relative efficiency of two- vs. one-stage production of astaxanthin by the green alga *Haematococcus pluvialis*. *Biotechnology and Bioengineering*. **2007**, *98*, 300-305.
22. Olaizola, M.; Huntley, M.E. Recent advances in commercial production of astaxanthin from microalgae, in Fingerman, M.; Nagabhushanam, R. (eds.), Recent Advances in Marine Biotechnology*. Volume 9. Biomaterials and Bioprocessing*. New Hampshire: Science Publishers, **2003**, 143-164.
27. Burczyk, J. Cell wall carotenoids in green algae which form sporopollenins. *Phytochemistry*, **1987**, *26*, 121-128.
42. Del Río, E.; Acién F.G.; García-Malea, M.C.; Rivas, J.; Molina-Grima, E.; Guerrero, M.G. Efficient one-step production of astaxanthin by the microalga *Haematococcus pluvialis* in continuous culture. *Biotechnology and Bioengineering*. **2005**, *91*, 808-815.
43. Torzillo, G.; Goksan, T.; Faraloni C.; Kopecky, J.; Masojidek, J. Interplay between photochemical activities and pigment composition in an outdoor culture of *Haematococcus pluvialis* during the shift from the green to red stage. *Journal of Applied Phycology*. **2003**, *15*, 127-136.
49. Holtin, K.; Kuehnle, M.; Rehbein, J.; Schuler, P.; Nicholson, G. Albert, K. Determination of astaxanthin and astaxanthin esters in the microalgae *Haematococcus pluvialis* by LC-(APCI) MS and characterization of predominant carotenoid isomers by NMR spectroscopy. *Analytical and Bioanalytical chemistry*, **2009**, *395 (6)*, p.1613.
56. Sarada, R.; Bhattacharya, S.; Ravishankar, G. Optimization of culture conditions for growth of the green alga *Haematococcus pluvialis*. *World Journal of Microbiology and Biotechnology*. **2002**, *18*, 517-521.
58. Dalay, M.C.; Imamoglu, E.; Demirel, Z. Agricultural fertilizers as economical alternative for cultivation of *Haematococcus pluvialis*. *Journal of Microbiology and Biotechnology*. **2007**, *17*, 393-397.
62. Oncel, S.S.; Imamoglu, E.; Gunerken, E.; Sukan, F.V. Comparison of different cultivation modes and light intensities using mono-cultures and co-cultures of *Haematococcus pluvialis* and *Chlorella zofingiensis. Journal of Chemical Technology and Biotechnology*. **2011**, *86 (3)*, 414-420.
77. Orosa, M.; Valero, J.F.; Herrero, C.; Abalde, J. Comparison of the accumulation of astaxanthin in *Haematococcus pluvialis* and other green microalgae under N-starvation and high light conditions. *Biotechnology Letters*, **2001**, *23* (13), 1079-1085.
78. Zhang, D.H.; Lee, Y.K. Ketocarotenoid production by a mutant of *Chlorococcum* sp. in an outdoor tubular photobioreactor. *Biotechnology Letters*, **1999**, *21*(1), 7-10.
79. Renstrøm, B.; Berger, H.; Liaaen-Jensen, S. Esterified, optical pure (3S, 3′ S)-astaxanthin from flowers of *Adonis annua*. *Biochemical Systematics and Ecology*, **1981**, *9* (4), 249-250.
80. Banerjee, K.; Ghosh, R.; Homechaudhuri, S.; Mitra, A. Biochemical composition of marine macroalgae from Gangetic Delta at the apex of Bay of Bengal. *African Journal of Basic & Applied Sciences*, **2009**, *1* (5-6), 96-104.
81. Yokoyama, A.; Adachi, K.; Shizuri, Y. New carotenoid glucosides, astaxanthin glucoside and adonixanthin glucoside, isolated from the astaxanthin-producing marine bacterium, *Agrobacterium aurantiacum*. *Journal of natural products*, **1995**, *58* (12), 1929-1933.
82. Bories, G.; Brantom, P.; de Barberà, J.B.; Chesson, A.; Cocconcelli, P.S.; Debski, B.; Dierick, N.; Franklin, A.; Gropp, J.; Halle, I.; Hogstrand, C. Safety and efficacy of Panaferd-AX (red carotenoid-rich bacterium (*Paracoccus carotinifaciens*) as feed additive for salmon and trout. *EFSA J.*, **2007**, *546*, 1-30.
83. Gassel, S.; Schewe, H.; Schmidt, I.; Schrader, J.; Sandmann, G. Multiple improvement of astaxanthin biosynthesis in *Xanthophyllomyces dendrorhous* by a combination of conventional mutagenesis and metabolic pathway engineering. *Biotechnology Letters*, **2013**, *35* (4), 565-569.
84. Zhang, X.W., Gong, X.D. and Chen, F. Kinetic models for astaxanthin production by high cell density mixotrophic culture of the microalga *Haematococcus pluvialis*. *Journal of Industrial Microbiology and Biotechnology*, **1999**, *23* (1), pp.691-696.
85. Hata, N.; Ogbonna, J.C.; Hasegawa, Y.; Taroda, H.; Tanaka, H. Production of astaxanthin by *Haematococcus pluvialis* in a sequential heterotrophic-photoautotrophic culture. *Journal of Applied Phycology*, **2001**, *13* (5), 395-402.
86. Kang, C.D.; Lee, J.S.; Park, T.H.; Sim, S.J.; 2005. Comparison of heterotrophic and photoautotrophic induction on astaxanthin production by *Haematococcus pluvialis*. *Applied Microbiology and Biotechnology*, **2005**, *68* (2), 237-241.
87. López, M.G.M.; Sánchez, E.D.R.; López, J.C.; Fernández, F.A.; Sevilla, J.F.; Rivas, J.; Guerrero, M.G.; Grima, E.M. Comparative analysis of the outdoor culture of Haematococcus pluvialis in tubular and bubble column photobioreactors. *Journal of Biotechnology*, **2006**, *123* (3), 329-342.

88. Ranjbar, R.; Inoue, R.; Shiraishi, H.; Katsuda, T.; Katoh, S. High efficiency production of astaxanthin by autotrophic cultivation of *Haematococcus pluvialis* in a bubble column photobioreactor. *Biochemical Engineering Journal*, **2008**, *39* (3), 575-580.
89. Kang, C.D.; Han, S.J.; Choi, S.P.; Sim, S.J. Fed-batch culture of astaxanthin-rich *Haematococcus pluvialis* by exponential nutrient feeding and stepwise light supplementation. *Bioprocess and Biosystems Engineering*, **2010**, *33* (1), 133.
90. Park, J.C.; Choi, S.P.; Hong, M.E.; Sim, S.J. Enhanced astaxanthin production from microalga, *Haematococcus pluvialis* by two-stage perfusion culture with stepwise light irradiation. *Bioprocess and Biosystems Engineering*, **2014**, *37* (10), 2039-2047.
91. Wan, M.; Zhang, Z.; Wang, J.; Huang, J.; Fan, J.; Yu, A.; Wang, W.; Li, Y. Sequential Heterotrophy–Dilution–Photoinduction Cultivation of Haematococcus pluvialis for efficient production of astaxanthin. *Bioresource Technology*, **2015**, *198*, 557-563.
92. Murphy, R.C.; James, P.F.; McAnoy, A.M.; Krank, J.; Duchoslav, E.; Barkley, R.M. Detection of the abundance of diacylglycerol and triacylglycerol molecular species in cells using neutral loss mass spectrometry. *Analytical biochemistry*, **2007**, *366* (1), pp.59-70.
93. Frassanito, R., Cantonati, M., Flaim, G., Mancini, I. and Guella, G. A new method for the identification and the structural characterisation of carotenoid esters in freshwater microorganisms by liquid chromatography/electrospray ionisation tandem mass spectrometry. *Rapid Communications in Mass Spectrometry*, **2008**, *22 (22)*, pp.3531-3539.
